# Antibacterial activity of a short de novo designed peptide against fish bacterial pathogens

**DOI:** 10.1007/s00726-024-03388-4

**Published:** 2024-04-05

**Authors:** Raja Aadil Hussain Bhat, Victoria C. Khangembam, Vinita Pant, Ritesh Shantilal Tandel, Pramod Kumar Pandey, Dimpal Thakuria

**Affiliations:** 1 ICAR-Directorate of Coldwater Fisheries Research, Bhimtal, 263136 Uttarakhand India; 2 Navsari Gujarat Research Centre, ICAR-Central Institute of Brackishwater Aquaculture, Navsari, 396 450 Gujarat India

**Keywords:** Antimicrobial peptide, De novo designing, Molecular docking, Bacteria, Antibacterial activity

## Abstract

In the face of increasing antimicrobial resistance in aquaculture, researchers are exploring novel substitutes to customary antibiotics. One potential solution is the use of antimicrobial peptides (AMPs). We aimed to design and evaluate a novel, short, and compositionally simple AMP with potent activity against various bacterial pathogens in aquaculture. The resulting peptide, KK12YW, has an amphipathic nature and net charge of + 7. Molecular docking experiments disclosed that KK12YW has a strong affinity for aerolysin, a virulence protein produced by the bacterial pathogen *Aeromonas sobria*. KK12YW was synthesized using Fmoc chemistry and tested against a range of bacterial pathogens, including *A. sobria, A. salmonicida, A. hydrophila, Edwardsiella tarda, Vibrio parahaemolyticus, Pseudomonas aeruginosa, Escherichia coli, Staphylococcus epidermidis*, and methicillin-resistant *S. aureus*. The AMP showed promising antibacterial activity, with MIC and MBC values ranging from 0.89 to 917.1 µgmL^−1^ and 3.67 to 1100.52 µgmL^−1^, respectively. In addition, KK12YW exhibited resistance to high temperatures and remained effective even in the presence of serum and salt, indicating its stability. The peptide also demonstrated minimal hemolysis toward fish RBCs, even at higher concentrations. Taken together, these findings indicate that KK12YW could be a highly promising and viable substitute for conventional antibiotics to combat microbial infections in aquaculture.

## Introduction

Global aquaculture production has been increasing steadily over the past several decades, and this growth trend is anticipated to persist in the upcoming years (Garlock et al. 2020). There are growing concerns about the possible threats to public health linked with the intensification of aquaculture systems (Heuer et al. 2009; Gormaz et al. 2014; Rodger 2016). This has been attributed to the increased incidence of novel infections and the growing dependence on antibiotics and other supplements (Bondad-Reantaso et al. 2005; Bhat et al. 2021, 2023). Studies have shown that these issues can lead to negative socioeconomic impacts for individuals and communities (Watts et al. 2017), with significant reductions in aquaculture productivity and commerce, amounting to over 6 billion USD annually (Mishra et al. 2012). Diseases are a major contributor to aquaculture production costs, accounting for anywhere between 10 and 50% of the total costs (Sahoo et al. 2017).

Antibiotics are regarded as the first line of defense to prevent and treat bacterial infections in aquaculture (Romero et al. 2012; Bhat et al. 2022a, b, c). The antibiotics are typically administrated as preventive and metaphylactic drugs, usually top coated on feed or administered directly to the aquatic environment (Cabello 2006; Pham et al. 2015; Okocha et al. 2018). Their widespread use in aquaculture has raised serious concerns due to the emergence of antibiotic-resistant bacteria, potential risks to food safety, and negative impacts on the environment (WHO 2006; Okocha et al. 2018). This has become a significant issue in many nations, as the indiscriminate use of antibiotics can lead to serious consequences for both human health and the environment. According to studies, 70–80% of antibiotics administered to fish are expelled into the water (Burridge et al. 2010; Cabello et al. 2013), resulting in aquaculture systems acting as "genetic hotspots" for gene exchange transfer (Watts et al. 2017). Thus, as our dependence on aquaculture increases, it is essential that we find viable antibiotic alternatives that may be delivered through feed or water, lower the risk of antimicrobial resistance developing, and boost fish immune systems. In the recent past, a number of methods that aim to promote sustainable aquaculture techniques have been proposed to control and manage the rise of antibiotic resistance, such as those involving the use of probiotics, vaccinations, and essential oils to boost fish immunological state (Sihag and Sharma 2012; Bhat et al. 2021). In recent times, natural AMPs are perceived as promising alternatives to antibiotics in aquaculture due to their ability to control and manage microbial infections without causing harm to the environment (Cárdenas et al. 2020; Chaturvedi et al. 2020; Valero et al. 2020; Bhat et al. 2022a, b, c).

AMPs are cationic, have broad-spectrum antibacterial action at low concentrations, and are not influenced by conventional causes of antibiotic resistance (Valero et al. 2020). The key characteristic of AMPs is their multifunctional method of action and non-toxic behavior toward mammalian cells due to their preferred interaction with microbial cells (Harris et al. 2009; Bahar and Ren 2013; Yasir et al. 2018; Bhat et al. 2022a, b, c). The appealing quality of AMPs is that they instantly and non-specifically target bacterial structures, notably membranes (Bahar and Ren 2013; Chaturvedi et al. 2020; Valero et al. 2020). Thus, there are less opportunities for the bacteria to undergo full membrane change and avoid numerous metabolic processes since AMPs might quickly damage the membrane and other vital internal components (Navon-Venezia et al. 2002). There is less possibility for bacteria to establish significant levels of resistance to AMPs as multiple mutations are not feasible for its survival (Navon-Venezia et al. 2002; Mookherjee and Hancock 2007).

However, despite their potential benefits, natural AMPs also have certain limitations as therapeutic molecules (Oyston et al. 2009; Vlieghe et al. 2010; Bhat et al. 2022a, b, c). AMPs are often rapidly degraded by proteases present in the host organism, reducing their effectiveness as therapeutic molecules (Oyston et al. 2009; Vlieghe et al. 2010). The artificial designing allows to modify these natural AMPs for improving their pharmacokinetics and pharmacodynamics properties, and making them more effective in treating infections with minimum toxicity (Bhat et al. 2020; Han et al. 2021; Bhat et al. 2022a, b, c). Moreover, synthetic AMPs can be modified to target specific pathogens by increasing their efficacy (Bhat et al. 2022a, b, c) and reducing the potential for resistance development. By artificially designing AMPs, all these properties may be combined in a single peptide molecule. At present, there are limited studies on the application of synthetic AMPs in aquaculture. To address this gap, the present research aimed to develop and synthesize a novel, short AMP with a simple composition using Fmoc chemistry. The KK12YW (KYWKKYWKKYWK-NH_2_) was analyzed using in silico tools and its antimicrobial efficacy was investigated by estimating MIC and MBC against common bacterial fish pathogens. Moreover, hemolytic and stability assays were done to evaluate the field applicability of the peptide. Additionally, molecular docking analysis of the KK12YW peptide with virulent protein of *A. sobria* was performed to investigate its binding mode and affinities. Further, bioinformatics approaches were employed to characterize the properties of the designed peptide.

## Materials and methods

### In silico designing and chemical synthesis of KK12YW peptide

The knowledge-based methodology was used to make the KK12YW peptide. Several noteworthy AMP physicochemical characteristics, such as net cationic charge, helicity, hydrophobicity, and amphipathicity, were taken into account during designing of the novel KK12YW peptide. These characteristics are essential for an AMPs to attach to a negatively charged bacterial membrane. The peptide was synthesized on rink amide MBHA resin (0.52 mmol. g^−1^) using Fmoc chemistry (Thakuria et al. 2017; Bhat et al. 2020; Bhat et al. 2022a, b, c). The purification of crude peptide was done by semi-preparative reversed phase-high performance liquid chromatography (RP-HPLC) (Shimadzu HPLC system, Japan) using water (A)/acetonitrile (B) gradient containing 0.1% trifluoroacetic acid (TFA) (Merck, Germany). The purification was performed with gradient conditions of 5% B initially to 95% B over 27 min and brought down to 5% within 3 min. Briefly, 500 μl of crude peptide (5 mg/ml in water) was injected into a chromolith monolithic SemiPrep RP-18e HPLC column (100–10 mm) (Merck, Germany). Flow rate was 3.0 ml/min and the chromatogram was monitored by UV absorbance at 220 nm. Further, the purity of the peptide was checked by RP-HPLC. The purified peptides were dissolved in nuclease free water (NFW). The mass of the peptide was confirmed using mass spectrometry.

### Bioinformatic analysis

We employed PepCalc.com (Lear and Cobb 2016) and HLP (Sharma et al. 2014) servers to determine the hydrophobicity and charge of KK12YW peptide at a neutral pH. In addition, protease cutting sites were predicted using PROSPER (Song et al. 2012), which helped to determine the stability of peptide in biological environments. Furthermore, PEP-FOLD 3 (Lamiable et al. 2016) was used to analyze the three-dimensional structures of peptide. The net wheels program (Mol et al. 2018) was employed to draw the helical wheel. The antibacterial activity of peptide was forecasted using multiple tools, such as CAMPR3 (Waghu et al. 2016) and APD3 (Wang et al. 2016). The RAMPAGE and saves v5.0 server was utilized to locate residues and evaluate the 3D structure quality in peptide. To predict the hemolysis potency of the designed peptide, the hemolytic web server was employed (Chaudhary et al. 2016).

### Molecular docking of KK12YW AMP with aerolysin of *A. sobria*

The crystal structure of *A. sobria* aerolysin in the Protein Data Bank (PDB) was not available. Thus, homology modeling of the *A. sobria* virulent protein was carried out using the SWISS-MODEL service, which utilizes a comparison of the submitted amino acid sequence to existing templates in the PDB (Arnold et al. 2006). The degree of similarity between the submitted sequence and the existing structures was used to construct the model. To improve the accuracy of the tertiary structure, the model was refined using the GalaxyRefine server (Ko et al. 2012). Furthermore, the quality of aerolysin 3D model was assessed using the RAMPAGE and ProCheck servers. The MDockPeP server was employed to conduct a blind docking study that involved the newly developed KK12YW peptide and the aerolysin protein of *A. sobria* (Xu et al. 2018). This process involved flexible docking of the peptide to the protein, which included creating conformations of the peptide and identifying potential binding modes on the protein’s surface. The most favorable docking poses were then selected based on their energy scores and further analyzed. The 3D structures and docking results of the peptide-protein complexes were visualized and analyzed using PyMOL, while LigPlot 2.1 was used for the 2D visualization of the ligand-receptor interactions (Laskowski and Swindells 2011).

### Determination of Inhibitory concentrations

#### Bacterial Strains, media and culture conditions

The study aimed to evaluate the effectiveness of a peptide against Gram-positive and Gram-negative bacteria as mentioned in Table 4. The bacterial strains were grown on nutrient agar at different temperatures depending on the specific bacterial species. For example*, Aeromonas hydrophila, A. sobria*, *Edwardsiella tarda, Pseudomonas aeruginosa* and were incubated at 30 °C, while *A. salmonicida was grown at* 20 °C for 12–14 h. Additionally, the growth of *Vibrio paraheamolyticus* was facilitated by adding 3% salt to the nutrient agar and broth. *Staphylococcus epidermidis*, Methicillin-resistant *S. aureus*, and *E. Coli* ATCC were incubated at 37 °C. To evaluate the effectiveness of KK12YW, individual colonies of each bacterial strain were carefully selected and cultured at their optimal temperature for 12 h. The bacterial cultures were then centrifuged, and the resulting pellets were suspended in normal saline to prepare a bacterial suspension with a concentration equivalent to a 0.5 McFarland standard solution.

#### Minimum inhibitory concentration (MIC)

To evaluate the antimicrobial activity of KK12YW peptide, microdilution assays were conducted according to CLSI guidelines (CLSI 2012) against a panel of Gram-positive and Gram-negative bacteria listed in Table 4. The bacterial suspension was adjusted to a cell density of 10^6^ CFU mL^−1^, and the antibacterial efficacy was evaluated at concentrations between 1834.21 and 0.45 µgmL^−1^ using a microtiter plate method. Two-fold serial dilutions were carried out up to the tenth well, followed by the addition of 50 µL of MH broth and 50 µL of the bacterial suspension. Two-fold serially diluted oxytetracycline was taken as positive control. After incubation for 16 h at the specified temperatures, 5 µL (6.7 mg mL^−1^) of resazurin dye was added to microtiter plate, followed by a 3-h incubation. The MIC value was taken as the lowest concentration at which a color remains blue (Sarker et al. 2007).

#### Minimum bactericidal concentration (MBC)

To test the effectiveness of a designed peptide in killing bacteria, the MBC was measured against various microorganisms. The bacterial suspension was first adjusted to a 0.5 McFarland standard and then diluted 1:100. Microtiter plates were used for the MBC assay, with bacterial suspensions of 10^6^ CFU mL^−1^added to achieve a final density of 5 × 10^5^ CFU mL^−1^. The plates were incubated at a specific temperature for 16 h, after which samples were taken from each well and streaked onto nutrient agar plates. Petri plates were then kept at the optimal temperature for each type of bacteria for 16 h and examined for any visible growth. The MBC was taken as the lowest concentration of peptide that prevented any visible growth. The experiment was performed in triplicate.

### Stability test of synthesized KK12YW AMP

The changes in the peptide’s MIC across different conditions were determined according to a specific protocol of Dong et al. (2018a, b) and Bhat et al. (2022a, b, c). The effectiveness of the AMP at high temperatures was assessed by subjecting it to various time intervals at 70 °C and 90 °C and determining its ability to inhibit the growth of *A. sobria*. The MIC value of the treated peptide was compared to that of the untreated peptide to determine any difference. Furthermore, the peptide’s tolerance different salt concentrations were assessed by subjecting it to varying levels of 150 mM and 650 mM NaCl, as well as 4.5 mM KCl.

### Serum stability assay

The designed AMP was subjected to different concentrations of fish serum dissolved in MHB, to evaluate the effect of protease enzymes. The fish serum concentrations used for this assessment were 25% and 50%. The MIC was determined according to guidelines from CLSI (CLSI 2012) as described previously. Additionally, we subjected KK12YW to trypsin treatment at a ratio of 100:1 (w/w), followed by an incubation period of one hour at 30 °C (Ebbensgaard et al. 2015). Subsequently, MIC of the trypsin-treated peptide was determined according to guidelines from CLSI (CLSI 2012).

### Binding of aerolysin with KK12YW peptide

To investigate the interaction between aerolysin and the KK12YW peptide, a hemolytic assay was conducted following the protocols outlined by Dong et al. (2017a, b). *Aeromonas sobria* strains stored at  – 80 °C were streaked on nutrient agar and incubated overnight. Subsequently, two to three colonies were selected and inoculated in nutrient broth until reaching a McFarland Standard 0.5 solution turbidity. The bacterial culture was inoculated in MHB with varying sublethal concentrations of the KK12YW peptide, ranging from 0.440 to 1.798 µgmL^−1^ for 16–18 h at 30 °C. After incubation, the culture tubes were centrifuged, and the resulting supernatant was collected in sterile tubes. Proaerolysin present in the supernatant was converted to aerolysin using 10 µg mL^−1^ trypsin (Iacovache et al. 2011). Subsequently, 50 μl of the trypsin-treated supernatant was co-incubated with 10% (v/v) fish RBCs in microtiter plates, with the untreated bacterial culture serving as a control. The microtiter plates were incubated at room temperature for 1 h, and unlysed cells were removed by centrifugation at 2500 × g for 7 min. Finally, hemoglobin release was quantified by measuring the absorbance of the supernatant at 540 nm. The hemolysis percentage (H) was calculated using the following formula:

$$H = \frac{{{\text{absorance}} {\text{of}} {\text{a}} {\text{sample}} - {\text{absorbance}} {\text{of}} {\text{blank}}}}{{{\text{absorbance}} {\text{of}} {\text{control}} - {\text{absorbance}} {\text{of}} {\text{blank}}}} \times 100$$

### Hemolytic assay against fish erythrocytes

The cellular toxicity of designed AMP was evaluated by quantifying the amount of hemoglobin released from common carp red blood cells (RBCs) using a spectrophotometer. Blood was drawn from the fish and RBCs were washed with a phosphate-buffered saline (PBS) solution, and then suspended in 10% (v/v) solution of PBS after centrifugation. Different concentrations of the AMP, ranging from 7336.84 to 0.440 µgmL^−1^, were added to the RBC solution, which was then incubated at 22 °C for 1 h. A control group was established by incubating RBCs with 0.2% Triton X-100, and a blank control was kept by incubating RBCs with PBS alone. The supernatant was collected from the incubated mixtures after centrifugation and transferred to a 96-well plate. The amount of hemoglobin released was determined by measuring the supernatant absorbance at 540 nm. The percent hemolysis was then calculated using a specific equation:

$$H = \frac{{{\text{absorance of}} {\text{a}} {\text{sample}} - {\text{absorbance}} {\text{of}} {\text{blank}}}}{{{\text{absorbance of}} 0.2\% {\text{triton}} X - {\text{absorbance}} {\text{of}} {\text{blank}}}} \times 100$$

A graph was plotted with the percentage of hemolysis caused by different peptide concentrations to determine the concentration of the peptide that resulted in 50% hemolysis (HC_50_) relative to the positive control (0.2% Triton X).

### Statistical analysis

A statistical analysis was conducted to determine the differences between treatment and control groups. The analysis was conducted using the one-way ANOVA method followed by Tukey’s multiple comparison test, and statistical significance was considered at *P* < 0.05. The data were obtained from five repetitions, and the mean and the standard error were calculated and reported. To indicate the significant differences between the treated and control groups, alphabets (a-i) were used and graphs were drawn using GraphPad Prism 8.

## Results

### Bioinformatic analysis designed peptide

The peptide was analyzed using various antimicrobial peptide prediction tools, such as the CAMPR3 database, which showed that the designed peptide had strong antimicrobial activity as shown in Table 1. Additionally, the iAMPpred tool also predicted that the peptide had properties of being antimicrobial, antiviral, and antifungal. The KK12YW peptide has a net positive charge of 7.0 with half of its amino acids being hydrophobic, and it is anticipated to fold into alpha helices with at least six lysine residues sharing a hydrophilic face. The peptide is composed of 50% lysine, 25% tryptophan, and 25% tyrosine. The physical and chemical properties were analyzed and listed in Table 2.

**Table 1:** The *In silico* analysis of antimicrobial activity of KK12YW peptide

S. no	Prediction tool	Probability score
1	Support vector machine	1.0
2	Random forest classifier	0.71
3	Artificial neural network classifier	1
4	Discriminant analysis classifier	0.93

**Table 2:** Physicochemical parameters of KK12YW peptide

S. no	Parameter	Value
1	Peptide sequence	KYWKKYWKKYWK- NH_2_
2	Molecular formula	C_96_H_131_N_21_O_16_
3	Hydrophobic ratio	25%
4	Stability	High
5	Hydrophobicity (KJ/mol)	17.150
6	pka	28.170
7	pKb	109.200
8	Molecular weight (g/mol)	1834.21
9	Isoelectric point	7.758
10	Charge	7.000
11	Polarity	308.130
12	Free Energy of solution (in water kcal/mol)	19.530
13	Extinction coefficient	20,910 M^−1^ cm^−1^
14	Estimated solubility	Good water solubility
15	Net Hydrogen	30.00
16	Amphipathicity index	4.83
17	GRAVY (i.e., the grand average hydropathy value of the peptide)	– 2.50
18	Protein-binding Potential	6.91 kcal/mol

**Fig. 1:**
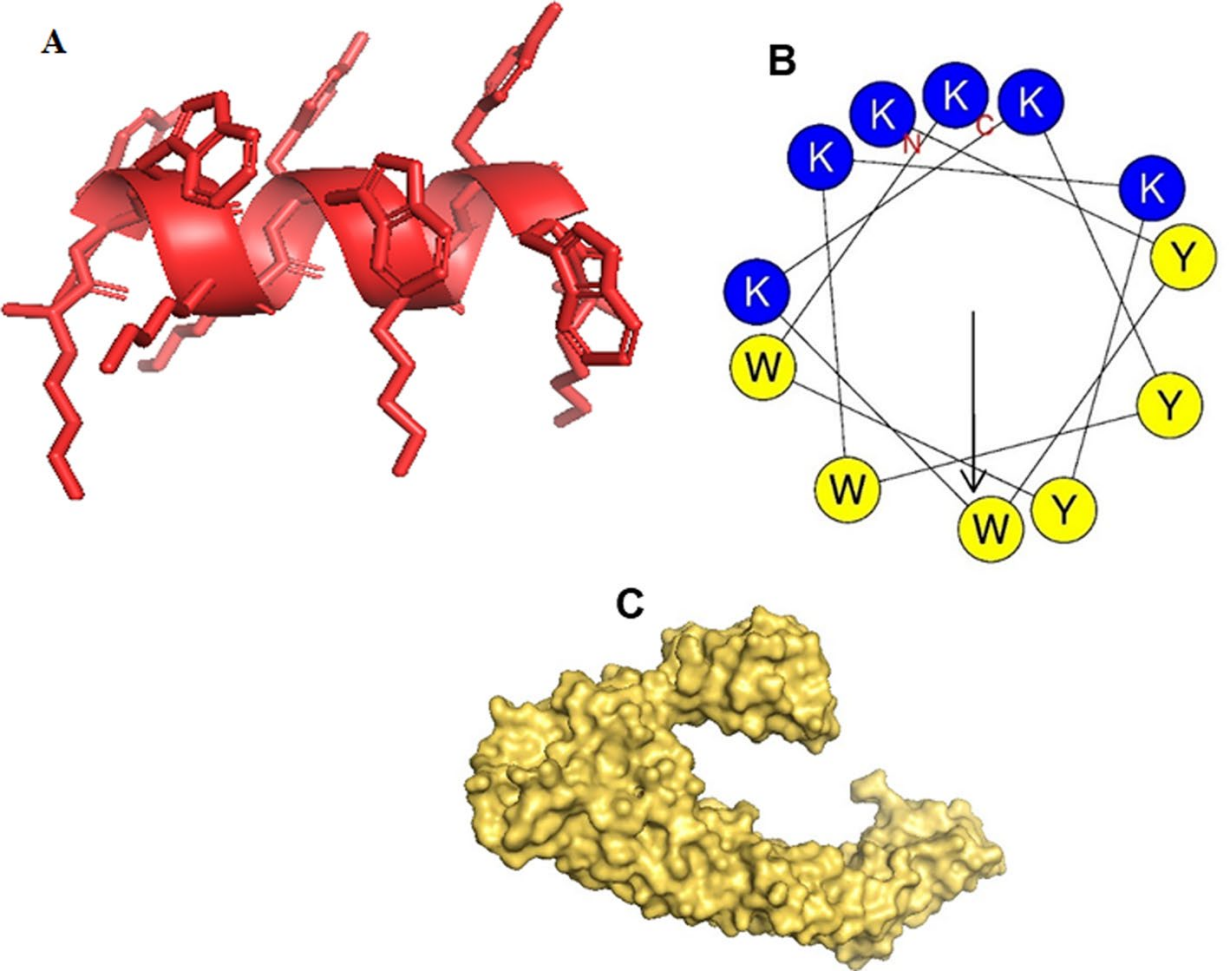
Tertiary structure (**A**), helical wheel (**B**) of the KK12YW AMP predicted by PEP- FOLD 3 server. C: tertiary structure of *A. sobria* aerolysin. The tertiary structures (3D) were drawn by PyMOL

The peptide’s favorable three-dimensional structure was confirmed using the SAVES server, which verified that all amino acids were located within the optimal region. In order to gain a better understanding of the helical structure of the peptide, as well as to analyze the polarity of its residues and intramolecular bonding, helical wheel was generated (Fig. 1). The helical wheel predicted that the peptide is amphipathic, as it contained both polar and nonpolar residues (Fig. 1B). Specifically, the helical wheel diagram illustrated the interactions between adjacent residues along the central axis of the peptide’s helix (Fig. 1B). Computational analysis also indicated that the peptide was robust against various types of proteases, including aspartic protease, cysteine protease, multiple proteases, and metalloprotease.

### Synthesis of the designed peptide

**Fig. 2:**
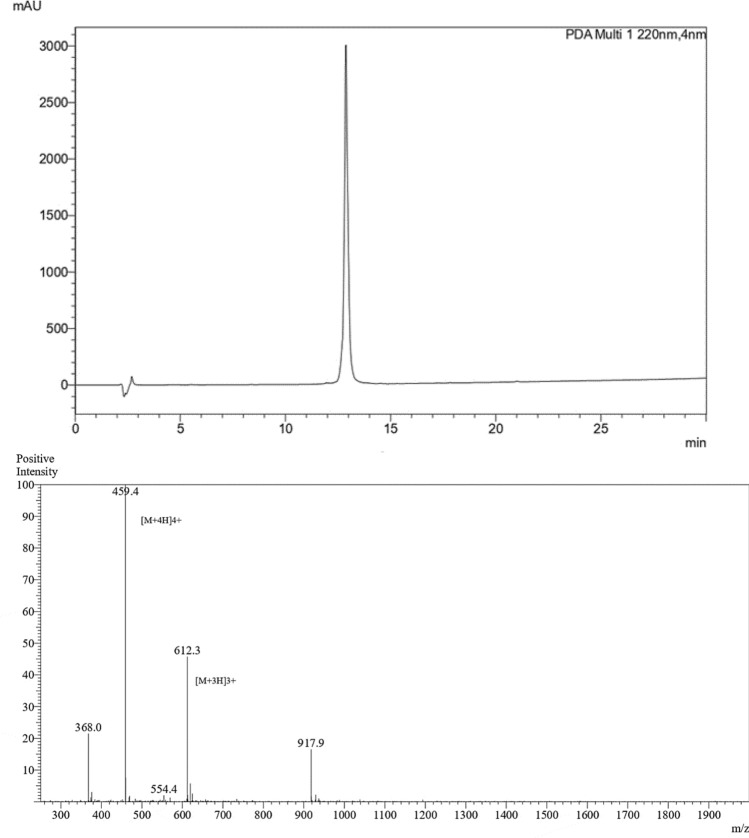
RP-HPLC chromatogram (top panel) and mass spectrum (below panel) of the KK12YW peptide

By employing the Fmoc chemistry approach, the peptide was synthesized and further purified via a Chromolith monolithic SemiPrep RP-18e HPLC column. The percentage yield of crude peptide was 75%. The purity percentage of peptide after HPLC purification was found to be 98%. Mass spectrometry was used to validate the peptide molecular weight, which corresponded with the calculated mass (Fig. 2 and Table 2).

### Molecular docking of KK12YW with aerolysin of *A. sobria*

**Fig. 3:**
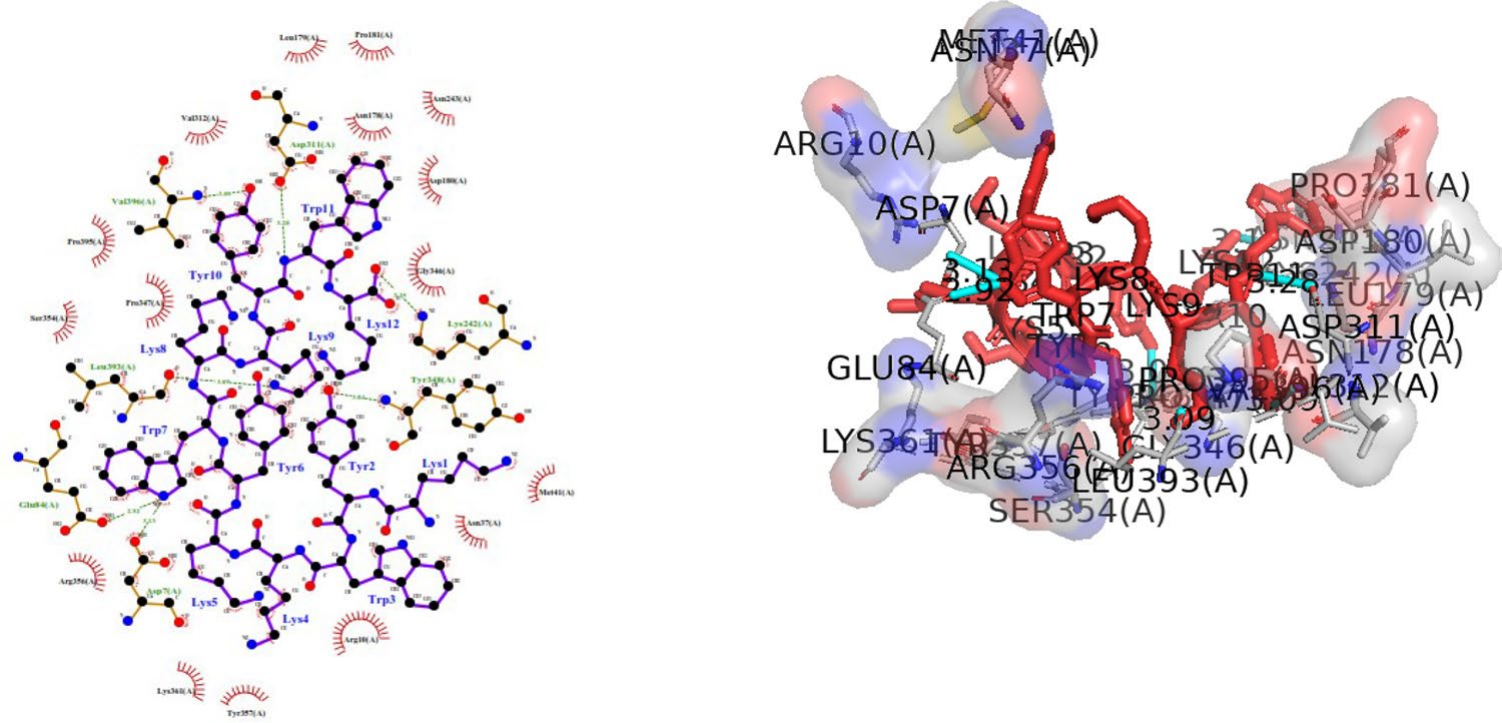
Amino acids of *A. sobria* aerolysin interacting with KK12YW AMP. Here left side of the figure depicts two-dimensional structure and right side shows tertiary structure of the aerolysin bound peptide. The peptide binds with the aerolysin of *A. sobria*, with the formation of seven hydrogen bonds and sixteen hydrophobic interactions. Here dotted green lines represent hydrogen bonding and red semi circles with spikes are hydrophobic interactions

The KK12YW peptide was computationally docked with the aerolysin protein of *A. sobria*. The interactions between the peptide and the aerolysin were analyzed and presented in Table 3 (Fig. 3). The results obtained from the MDockPeP server using blind docking method, revealed that the peptide effectively binds to the virulence protein of *A. sobria*, with the formation of seven hydrogen bonds and sixteen hydrophobic interactions between the bound complex.

**Table 3:** Hydrogen bonding interaction between amino acids (AA) of KK12YW peptide and aerolysin of *A. sobria*

S.no	KK12YW AA	Aerolysin AA	Hydrogen bond (˚A)
	TYR 10	VAL396	3.087
	LYS 9	LEU 393	3.086
	TYR2	TYR 348	3.041
	TRP 11	ASP 311	3.284
	LYS 12	LYS 242	3.154
	TRP 7	GLU 84	2.919
	TRP 7	ASP 7	3.128

### Inhibitory values of designed AMP against selected pathogens

The MIC and MBC values obtained against selected bacterial pathogens are listed in Table 4. The results revealed that the KK12YW peptide exhibited antimicrobial activity against gentamicin and methicillin-resistant *S. aureus, E. tarda, A. sobria, V. paraheamolyticus, P. aeruginosa, E. coli,* and *S. epidermidis* at relatively low concentrations While the peptide also showed effectiveness against *A. salmonicida*, but a higher concentration was required for the same. The peptide demonstrated bactericidal activity against all tested bacterial strains, with MBC values that were less than four times their respective MIC values, except for *A. sobria* and *P. aeruginosa*.

**Table 4:** Antibacterial activity parameters (MIC and MBC) of KK12YW peptide. OTC was taken as control.positive

Bacteria	MIC (µgmL^−1^)	MBC (µgmL^−1^)	MIC (µgmL^−1^) of OTC
*A. sobria* MTCC 3613	0.89	3.67	0.19
*A. hydrophila* fish isolate ampicillin resistant	458.56	550.26	0.78
*A. salmonicida* (NCBI GenBank accession No. MN093875.1)	917.10	1100.52	2
Gentamicin and Methicillin-resistant *S*. *aureus* ATCC 33592	57.32	64.19	0.98
*E. Coli* ATCC 8739	28.79	33.01	0.19
*P. aeruginosa* ATCC 9027	0.89	14.67	0.19
*E. tarda* ATCC 15947	114.63	330.16	0.097
*S. epidermidis* ATCC 35984	7.171	18.34	0.097
*V. parahaemolyticus* ATCC 17802	114.63	137.56	0.097

### Stability assay

To assess the effectiveness of KK12YW against *A. sobria*, the peptide underwent thermal and salt stress by subjecting it to preheating at 70 °C and 90 °C for varying periods. The findings revealed that even after exposure to elevated temperatures, the designed KK12YW maintained its ability to prevent microbial growth, with no significant changes in the MIC values compared to the control group (Table 5). Furthermore, the antimicrobial activity of the peptide against *A. sobria* remained unaffected by the presence of potassium and sodium cations (Table 5). The peptide was exposed to increasing serum concentrations, its MIC values showed an increase (as shown in Table 5). Following trypsin treatment, the peptide exhibited a MIC value of 57.32 µgmL^−1^ against *A. sobria*. Nonetheless, the KK12YW peptide persisted in its capacity to destroy microorganisms even at higher serum concentrations.

**Table 5:** MIC (µgmL^−1^) of KK12YW at higher temperatures and in different physiological conditions

Peptide	Thermal Stability						Salt Stability			Serum Stability	
	70ºC			90ºC			NaCl (150 mM)	NaCl (650 mM)	KCl (4.5mM)	25%	50%
	0 min	15 min	30 min	0 min	15 min	30 min					
KK12YW	0.898	0.898	0.898	0.898	0.898	0.898	0.807	0.807	1.632	7.15	14.32

### Aerolysin binding with peptide

**Fig. 4:**
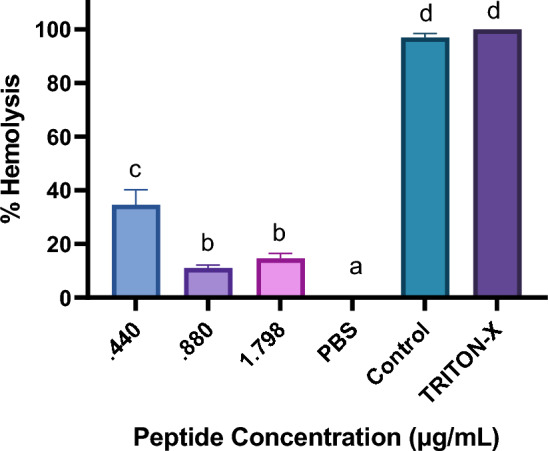
Hemolytic activities of the *A. sobria* supernatants co-incubated with sub lethal concentrations of KK12YW peptide. The untreated bacterial culture was taken as control and Triton X was taken as positive control and PBS as negative control

At sub-inhibitory concentrations, KK12YW peptide inhibited the hemolytic activity of *A. sobria* culture supernatants. When treated with 0.440, 0.880, and 1.798 µgmL^−1^, the percentages of hemolysis were 34.63%, 11%, and 14.66%, respectively (Fig. 4).

### Hemolytic assay

**Fig. 5:**
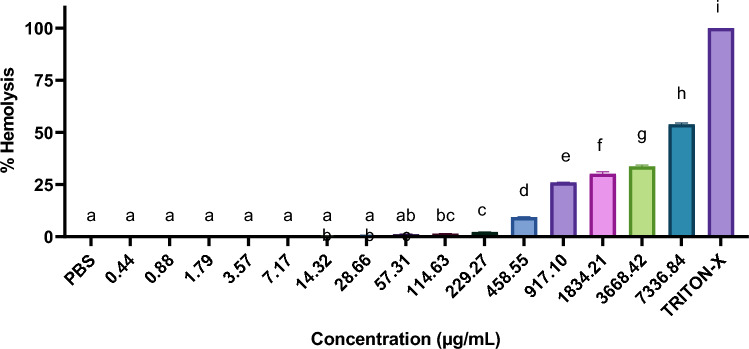
Hemolytic activity of KK12YW peptide. Here Triton X was taken as positive control and PBS as negative control

The KK12YW peptide caused 51.2% hemolysis in fish RBCs at 7336.84 µg mL^−1^ (Fig. 5). Additionally, the hemolytic database revealed that the designed AMP was non-hemolytic with a hemolytic score of 0.

## Discussion

There is currently a dearth of research that investigates the efficacy of artificially designed synthetic AMPs in fighting bacterial infections in aquaculture. This study aims to address this gap by presenting the rational design of a short and novel 12-mer AMP that strikes a balance between its antimicrobial activity and cytotoxicity. The AMP was intentionally created to have a unique structure when folded into an alpha-helix, with one side comprised of hydrophobic residues and the other composed of hydrophilic residues. This particular structure, referred to as an amphipathic alpha-helix, is crucial for effective interactions with microbial membranes through both electrostatic and hydrophobic mechanisms, leading to its antibacterial properties. (Zelezetsky and Tossi 2006; Chaturvedi et al. 2020).

The KK12YW AMP has been designed with specific amino acids, such as lysine and tryptophan, because of their unique characteristics. Lysine, with its amine groups in the side chain, interacts with negatively charged phosphatidylglycerol found in the plasma membrane of bacteria or lipopolysaccharides of Gram-negative bacteria and lipoteichoic acid of Gram-positive bacteria (Killian and von Heijne 2000). Additionally, the long aliphatic side chain of lysine plays a role in the peptide’s localization in the lipid bilayer of the plasma membrane (Segrest et al. 1990; de Planque et al. 1999; Killian and von Heijne 2000). Tryptophan, on the other hand, interacts with the polar–apolar interface through its indole ring (Killian and von Heijne 2000; Hong et al. 2013; Sparks et al. 2014). Tyrosine, which has a hydroxyl group on its aromatic ring, is well-suited for interactions at the interfacial regions of membrane proteins (Killian and von Heijne 2000). Reports indicated that AMPs exhibit their highest biological effectiveness within a charge range of + 4.3 to + 7 (Tossi et al. 2000; Giangaspero et al. 2001). AMPs have the ability to form a strong electrostatic bond with bacterial membranes by binding to the interface of lipid bilayer (Yount et al. 2006). The presence of amino acids with a positive charge and overall cationicity is crucial for the antimicrobial activity of AMPs (Tossi et al. 2000; Dathe et al. 2001; Giangaspero et al. 2001). In order to enhance the peptide’s antimicrobial properties, an amide group was added to its C-terminus. This alteration affects the distribution of positive charges and modifies certain physical and chemical characteristics, ultimately improving the peptide capacity to interact with bacterial cell membranes and increasing its antimicrobial effectiveness properties (Zasloff 2002; Bhat et al. 2022a, b, c). Previous research has highlighted the critical role of amidation in making lytic peptides effective against bacteria (Zasloff 2002; Bhat et al. 2022a, b, c). In agreement with previous studies, our study indicates that the addition of an amide group at the C-terminus of the peptide improved its structural integrity and antibacterial activity while also minimizing its proteolytic activity (Dennison and Phoenix 2011; Bhat et al. 2020; Bhat et al. 2022a, b, c). Moreover, such modifications have been shown to impede the proteolytic activity in AMPs as observed in cecropin (Moore et al. 1994). The isoelectric point (pI) of the designed peptide was kept close to neutral pH (around 7) which is generally convenient for working with peptides, as they tend to be more soluble in water at or near their pI. This is often desirable for biological and biochemical studies (Hamley 2020). Another important parameter, GRAVY is determined by dividing the sum of hydropathy values of all amino acids by the number of residues (Bisana et al. 2013). This score ranges from  – 3 to + 2, where a negative score indicates hydrophilicity, and a positive score indicates hydrophobicity. GRAVY score of our peptide is  – 2.50. Peptides with a more negative GRAVY score are considered to be hydrophilic in nature hence good solubility and better bioavailability (Roshanak et al. 2023).

After verifying the structure and assessing its ability to combat microbes via computational methods, molecular docking procedure was executed against aerolysin, a crucial virulence protein present in *A. sobria*. This particular protein is a critical virulence factor in *A. sobria* and is responsible for the destruction of red blood cells, leading to severe blood infections in fish (Dong et al. 2017a, b; Lian et al. 2020). As a result, aerolysin has been recognized as a promising and robust target in the development of new pharmaceuticals by inhibition of its function fish (Dong et al. 2017a, b; Lian et al. 2020). Several authors have effectively discovered small molecules and antibodies that effectively inhibit the activity or expression of aerolysin (Dong et al. 2017a, b, 2018a). In this particular study, we found that the KK12YW peptide forms strong hydrogen bonds and hydrophobic interactions with aerolysin, leading to the inhibition of its activity and weakening of the virulence of *A. sobria*. Furthermore, the docking analysis and the identification of the specific amino acids involved in the binding process suggest that the KK12YW peptide has an ideal binding that is consistent with the results obtained from laboratory experiments. Thus, we propose that the peptide binds to the cell wall, facilitated by the robust hydrogen bonds and hydrophobic interactions, leading to the suppression of aerolysin. Overall, these findings suggest that the KK12YW peptide could potentially serve as a novel therapeutic agent for combating *A. sobria* infections, providing a promising avenue for future research in the field of antimicrobial drug development.

The newly designed peptide has demonstrated its effectiveness in eliminating antibiotic-resistant strains of *A. hydrophila* and *A. salmonicida*, which indicates its potential as a viable solution for the treatment of hemorrhagic septicemia and furunculosis in aquaculture. Furthermore, the peptide showed promising activity against *E. tarda* at low concentrations, suggesting its potential use in the treatment of edwardsiellosis in fish. The broad-spectrum antimicrobial activity of peptides makes it a promising candidate for treating various microbial infections in aquaculture. Additionally, several researchers have investigated the efficacy of AMPs against human pathogens (Waghu et al. 2016; Vergis et al. 2019). There is a notable dearth of available reports on the utilization of artificially designed AMPs in aquaculture. In this line, Leon et al. (2020) conducted experiments to assess the effectiveness of various natural AMPs against bacterial pathogens affecting fish. To their surprise, none of the tested peptides demonstrated any antimicrobial activity against *A. salmonicida*. Similarly, Choi et al. (2020) found that the MIC of two short AMPs derived from *Platichthys stellatus* ranged from 62.5 to over 500 µgmL^−1^against several fish pathogens. In a related study, Chen et al. (2020) reported that Topmouth culter LEAP-2 exhibited inhibitory effects on aquatic bacterial growth, including antibiotic-resistant bacteria, with MIC values ranging from 18.75 to 150 µgmL^−1^. Likewise, Jia et al. (2000) documented that two highly effective AMPs, namely CEME (a cecropin-melittin hybrid peptide) and pleurocidin amide (a C-terminally amidated form of the natural flounder peptide), have the potential to protect coho salmon from *V. anguillarum* infections. These findings collectively underscore the ongoing exploration of AMPs in aquaculture and their varied impacts on different fish pathogens.

The differences in MIC values of AMPs can be attributed to variations in the levels of essential bacterial membrane components, such as phosphatidylethanolamine, phosphatidylglycerol, and cardiolipin, which can affect the interaction ability of AMPs (Epand and Epand 2009). Furthermore, important consideration in the variations of MIC values among AMPs is their differential binding preferences and specificity to various bacterial strains (Epand and Epand 2009; Mishra et al. 2012). AMPs possess broad-spectrum antimicrobial activity, but their biological application is restricted by their vulnerability to proteolytic degradation within the biological system and their relatively high cellular toxicity (Han et al. 2020). The AMP examined in our study demonstrated the ability to withstand high temperatures, which makes it a promising candidate for applications in industries, such as food processing and packaging, where high temperatures are frequently utilized.

Our investigation has revealed that the newly developed AMP retained its antimicrobial efficacy even when tested under various physiological conditions, including 150 & 650 mM NaCl and 4.5 mM KCl. This indicates its high stability in the presence of different physiological salts, making it a promising option for the treatment of microbial infections in aquaculture. Despite a decrease in antimicrobial activity observed at high serum concentrations, the AMP still demonstrated significant potential against the tested bacteria. Moreover, our findings are consistent with the in silico predictions, which suggest that the designed AMP possesses a high resistance to proteases, thus minimizing the likelihood of in vivo enzymatic degradation (Ma et al. 2012). In addition to conferring antimicrobial activity, the consideration of low cytotoxicity in the design of AMPs is of utmost importance as it can significantly influence their field application. Our research has successfully demonstrated that the designed peptide exhibits minimal hemolytic activity even at high concentrations. These results align with the predictions obtained through hemolytic assays, which further indicate to the peptide’s non-hemolytic nature. Moreover, our study has established that the HC_50_ of the designed AMP is higher than its MIC and significantly lower in comparison to other AMPs previously designed by other researchers (Qi et al. 2010; Ebbensgaard et al. 2015), Consequently, these observations unequivocally indicate that the designed AMP holds considerable promise as a potent antimicrobial agent in the field of aquaculture.

## Conclusions

We have developed a novel and compositionally simplistic KK12YW AMP, which possesses an amphipathic sequence. This AMP was constructed using a knowledge-based methodology and has demonstrated to exhibit potent antimicrobial activity against typical fish pathogens. Notably, the AMP showed minimal cytotoxicity toward fish erythrocytes, making it a promising candidate for use as an antimicrobial agent. Furthermore, the AMP exhibited broad-spectrum antimicrobial activity against diverse bacterial strains, including antibiotic-resistant strains, such as *S. aureus, A. hydrophila*, and *A. salmonicida*, indicating its potential as an effective anti-infective agent for aquaculture. Importantly, the AMP displayed high thermal stability, making it suitable for use in the food and feed processing industry. Overall, our findings suggest that the designed AMP holds potential as a potent and safe antimicrobial agent for aquaculture and other industries. The simple peptide composition and its potent antimicrobial activity make it an attractive candidate for further development and exploration.

## Data Availability

The data of this study are available within the article.
